# Reviewing neonicotinoid detection with electroanalytical methods

**DOI:** 10.1007/s11356-024-33676-1

**Published:** 2024-05-21

**Authors:** Bartłomiej Barton, Nabi Ullah, Kamila Koszelska, Sylwia Smarzewska, Witold Ciesielski, Dariusz Guziejewski

**Affiliations:** Department of Instrumental Analysis, University of Lodz, Pomorska 163, 90-236 Lodz, Poland

**Keywords:** Voltammetry, Electroanalysis, Neonicotinoid, Determination, Pesticide, Electrochemical analysis

## Abstract

Neonicotinoids, as the fastest-growing class of insecticides, currently account for over 25% of the global pesticide market. Their effectiveness in controlling a wide range of pests that pose a threat to croplands, home yards/gardens, and golf course greens cannot be denied. However, the extensive use of neonicotinoids has resulted in significant declines in nontarget organisms such as pollinators, insects, and birds. Furthermore, the potential chronic, sublethal effects of these compounds on human health remain largely unknown. To address these pressing issues, it is crucial to explore and understand the capabilities of electrochemical sensors in detecting neonicotinoid residues. Surprisingly, despite the increasing importance of this topic, no comprehensive review article currently exists in the literature. Therefore, our proposed review aims to bridge this gap by providing a thorough analysis of the use of electrochemical methods for neonicotinoid determination. In this review article, we will delve into various aspects of electrochemical analysis, including the influence of electrode materials, employed techniques, and the different types of electrode mechanisms utilized. By synthesizing and analysing the existing research in this field, our review will offer valuable insights and guidance to researchers, scientists, and policymakers alike.

## Introduction

Over the last century, there has been an incredible increase in the human population. Since 1915, it has increased fourfold. This surge led to the milestone of 8 billion people being reached on November 15, 2022. It is estimated that by 2080, the population will exceed 10 billion. However, with population growth comes an inevitable increase in global food production. By 2050, the demand for food is expected to increase from 59% to almost 100%. As a result, new methods have been explored for years to increase the efficiency of food production and cultivation. One such method is the use of crop pest control agents. However, the continuous development of resistance in insects, global restrictions on using environmentally friendly agents, and population growth highlights the need for safer and more effective insecticides (Crossthwaite et al. 2017; Chamie 2022; Maarten and Florian 2016).

Nicotine was originally used as an insecticide to control pests in crops because of its powerful effects on nicotine acetylcholine receptors (nAChR) situated in the insects’ central nervous system (CNS). Its insecticidal efficacy relied on nicotine penetrating the CNS, acting on a specific receptor directly in the synapse. In mammals, nAChR receptors are present in both the central nervous system and the peripheral nervous system (PNS). nAChR receptors in the PNS are easily accessible to nicotine, resulting in acute intoxication. Due to nicotine’s toxic effect on the PNS in mammals, it was phased out and replaced with neonicotinoids (Yamamoto 1999).

Neonicotinoids are at the top of the most important chemical class of compounds used in plant protection against insects. They have been registered in over 120 countries worldwide. Despite a slight decrease (30% in 2012), holding a 24% share of the global market means they remain the most commonly used pesticides. Combined sales of all nicotine acetylcholine receptor pesticides amounted to over $4 billion in 2014, with neonicotinoids alone accounting for $3.3 billion (83%) (Crossthwaite et al. 2017; Bakker et al. 2020; Sparks and Lorsbach 2017). In recent years, due to the negative impact of imidacloprid on the environment, its use has decreased. However, there has been an increase in the use of compounds such as clothianidin, thiamethoxam, and acetamiprid. In the coming years, the share of neonicotinoids in the global market is expected to increase (Zhang et al. 2023a).

**Fig. 1:**
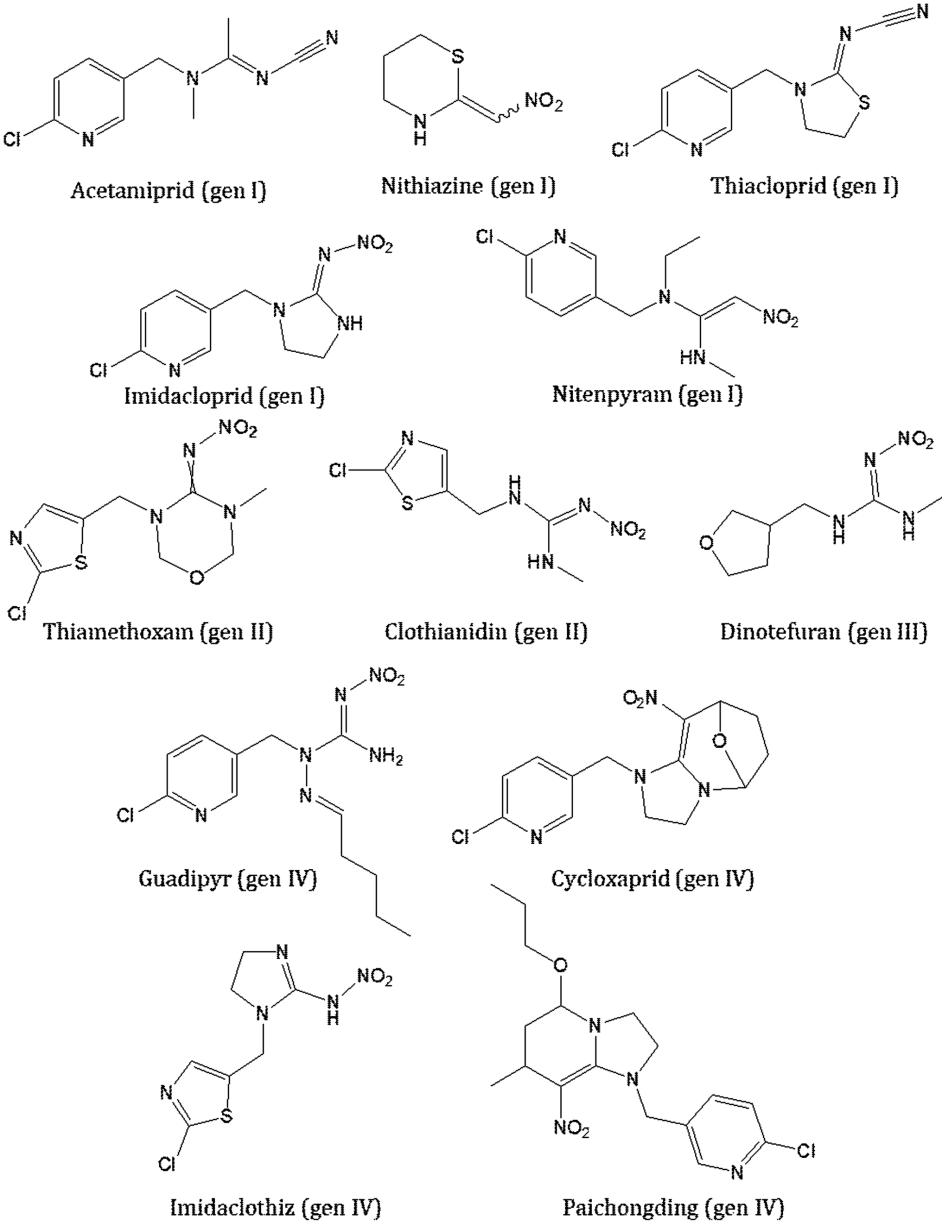
Chemical structure of neonicotinoids and their generation class assignment

Neonicotinoids are a class of compounds recognized as neuroactive pesticides, which quickly gained popularity due to their specific features. They are notable for their high water solubility and their slow decomposition in soil, enabling plants to absorb them more efficiently. Furthermore, the production of neonicotinoids is much cheaper than that of nicotine, and therefore, more profitable for industry (Buszewski et al. 2019). They consist of compounds belonging to the first generation: nithiazine, acetamiprid, imidacloprid, nitenpyram, and thiacloprid; the second generation: clothianidin and thiamethoxam; the third generation: dinotefuran; and the fourth generation: cycloxaprid, imidaclothiz, guadipyr, and paichongding (Thompson et al. 2020) (Fig. 1). These compounds target the nAChR of insects (Lezi and Economou 2015; Crossthwaite et al. 2017). Bayer CropScience introduced the nicotinoid precursor, imidacloprid, in 1991 to protect plants from pests (Jeschke et al. 2011). These compounds are derivatives of nicotine and consequently target specific receptors in the insect’s CNS. While vertebrates also possess nAChR receptors, their affinity for neonicotinoids is significantly lower than that of invertebrates. This means that these compounds are far more harmful to insects than to other animals, such as mammals and birds (Zhang et al. 2023b). The greater toxicity of neonicotinoids towards insects is mainly due to the selectivity of the substance towards a specific nAChR receptor subtype (α4β2 in insects) as well as the lack of a blood–brain barrier. Nonetheless, despite the relatively low toxicity of neonicotinoids to humans, these substances may still pose a danger. According to research by Selvam and Srinivasan (2019), severe poisoning in humans may mainly result from oral ingestion of neonicotinoids. Penetration through the skin upon contact has not been extensively studied quantitatively, and due to the low volatility of neonicotinoids, their absorption through the respiratory system is minimal. The clinical outcomes and toxicokinetic of imidacloprid following self-poisoning in humans were investigated by Mohamed et al. (2009) and Forrester (2014). According to these studies, imidacloprid exhibits a low mortality rate in humans, despite consumption even in large amounts. The predominant symptoms reported include nausea, headache, vomiting, and diarrhoea. Imidacloprid poisoning induces continuous stimulation of nAChR receptors, leading to nerve transmission blockage. Consequently, fatigue occurs, accompanied by convulsions, severe headaches, disorientation, drowsiness, decreased muscle tone, and coma. Severe clinical manifestations such as renal failure, respiratory failure, and ventricular fibrillation were less frequently reported. Only one case of patient fatality due to organ failure following combined neonicotinoid and alcohol poisoning has been documented (Yeh et al. 2010).

Neonicotinoids can be classified in several ways. The neonicotinoid class includes various compounds, which can be divided into subgroups based on the molecular structure of the compounds. When considering the chemical structure of these aromatic heterocyclic compounds, the most common way to classify them is the presence of conjugated bonds. Internally, the molecular structure of neonicotinoids can be divided into four distinct sections: aromatic heterocyclic, flexible linkage, hydroheterocycle or guanidine/amidine, and electron-withdrawing substitution (Buszewski et al. 2019; Yang et al. 2014). Depending on their molecular structure, neonicotinoids can be categorized into specific groups: N-nitroguanidines and N-cyanamidines. N-nitroguanidines possessing the N-nitro group—and consequently an oxygen atom—show greater chemical activity and polarity. The compounds of this group are more reactive. Examples of compounds from this group include imidacloprid, thiamethoxam, nitenpyram, dinotefuran, nithiazine, and clothianidin. On the other hand, compounds from the cyanamide group, such as acetamiprid and thiacloprid, lack oxygen atoms in their structures. As a result, they are less reactive than their oxygen-containing counterparts. Their higher seed degradation rate restricts their use in controlling seed insects (Buszewski et al. 2019).

Despite their differences, the target site of action for all neonicotinoids is the nicotinic nAChR receptor. Hence, when considering their mechanism of action, these compounds are viewed as functionally identical. This unified effect, acting as agonists of the acetylcholine receptors, is attributed to their shared active moiety. However, this does not imply that all neonicotinoids exhibit the same properties. Individual compounds differ from each other in susceptibility to decomposition in the soil, as well as the impact they have on the metabolism of any insects and especially pollinating insects, e.g. bees (Buszewski et al. 2019).

Neonicotinoids exhibit their wide application in insect control, establishing themselves as the most frequently used class of insecticides (Klingelhöfer et al. 2022). In the USA alone, over 1000 products containing neonicotinoids are officially registered (Thompson et al. 2020). They have been used in many agricultural crops (Table 1), including cotton, corn, rice, potato, and rapeseed. These compounds protect crops against various insects such as corn rootworms, white grubs, and wireworms (Klingelhöfer et al. 2022). As systemic insecticides, neonicotinoids are readily absorbed by plants and transported throughout the body. This property is used to ensure the long-term protection of plants, starting from seed purification, which later transports substances through the stems and leaves to pollen and nectar (Buszewski et al. 2019). However, crops are not the only function of neonicotinoids. These nicotine derivatives also serve in home, lawn, and garden products, combating pests like termites, ants, and cockroaches. Additionally, neonicotinoids have found utility in veterinary medicine, effectively tackling fleas and ticks on pets such as dogs and cats (Klingelhöfer et al. 2022, Craddock et al. 2019).

**Table 1:** Neonicotinoids and examples of their applications (Buszewski et al. 2019; Elbert et al. 2008)

Active substance	Application
Acetamiprid	Protection of agricultural plants such as potatoes, beets, oilseed rape, and tobacco, as well as fruit and vegetable plants such as apple, raspberry, blackcurrant, vines, strawberry and onion, tomato, pepper, and cucumber. Used to control termites and domestic pests
Clothianidin*	Used on seeds and leaves. Protects crops of rice, cereals, corn, rapeseed, fruit, potatoes, sugar beets, and vegetables
Dinotefuran	Control of sucking insects on vegetables, fruits, sugar beets, rice, and cotton
Imidacloprid*	Fighting running, flying, and sucking insects such as cockroaches, flies, and mosquitoes. Protects major crops such as cereals, cotton, rice, sugar, rapeseed, vegetable, and ornamental crops. Protecting animal health as well as lawns and gardens
Nitenpyram	For the treatment of cat fleas and sucking insects in rice, fruit, tea, and vegetables
Thiacloprid	Protection of cotton, vegetables, rape, grain, and potatoes against sucking and chewing insects. Safe for bees and pollinating insects
Thiamethoxam*	Protection of potatoes, rice, cotton, fruit, tobacco, and grain. Used to control all sucking insects as well as some chewing and soil-dwelling insects, e.g. potato beetles and cactus cotton

^*^Prohibited for use in field crops in 2018 in Europe (Stokstad 2018)

The frequent use of neonicotinoids as insecticides has led some insect species to develop resistance (Buszewski et al. 2019). Therefore, the development of new neonicotinoid compounds has become essential to effectively combat them. To address this, new substances have been developed by modifying the moieties mentioned above, such as introducing a functional sulfonylamide group that replaces cyano- or nitro-guanidine/amidine (Buszewski et al. 2019; Yang et al. 2014). As a result, compounds classified as fourth-generation neonicotinoids and neonicotinoid-like have been created. These compounds include guadipyr, cycloxaprid, paichongding, imidaclothiz, flonicamid, flupyradifurone, and sulfoxaflor. Of these, the last three are sometimes considered neonicotinoids, but only flonicamid and flupyradifurone possess a neonicotinoid-like mechanism of action. The registration for guadipyr, cycloxaprid, paichongding, and imidaclothiz declined in both Europe and the USA (Bas et al. 2017). It is worth noting that over 600 new neonicotinoid compounds have been synthesized in China recently (Bas et al. 2017; Thompson et al. 2020).

The low toxicity to mammals contributed to such a wide use of these insecticides. It is estimated that neonicotinoids are 5–10 times more selective against insects than organophosphates (Klingelhöfer et al. 2022). However, neonicotinoids are not so selective for all needed animals. Some are highly toxic to pollinating insects, especially honeybees (Table 2). This is especially true for neonicotinoids that have an N-nitro group in their structure. While neonicotinoids do not directly kill bees, they result in a concerning mortality rate. Exposure to neonicotinoids can lead to various behavioural disorders in honeybees, such as difficulties in orientation, impaired social skills, and memory problems (Laurino et al. 2011).

**Table 2:** Selected properties of neonicotinoids (Borsuah et al. 2020, European Food Safety, A, 2012, Aquatic Life Benchmarks and Ecological Risk Assessments for Registered Pesticides 2022, Wood and Goulson 2017)

Compound	Solubility in water (20 °C) (g/L)	Toxicity limit in freshwater for fish (mg/L)	Acute oral toxicity LD_50_ (μg/bee)	Acute contact toxicity LD_50_ (μg/bee)
Acetamiprid	2.95	> 50	14.53	8.09
Imidacloprid	0.61	114.5	0.0037	0.081
Nitenpyram	590	–	–	–
Thiacloprid	0.184	12.6	17.32	38.82
Clothianidin	0.340	> 50.75	0.00379	0.04426
Thiamethoxam	4.1	> 57.70	0.005	0.024
Dinotefuran	39 830	> 49 500	–	–

In response to the increasing concerns about the impact of neonicotinoid insecticides on pollinators and the environment, several countries have taken significant measures to restrict or ban their use. For example, the European Union imposed a complete ban on the outdoor use of three neonicotinoids—clothianidin, imidacloprid, and thiamethoxam—on flowering crops in 2018. This ban was expanded in 2021 to include all outdoor uses of these neonicotinoids. Canada, France, Germany, and the UK have also implemented restrictions to protect pollinators and biodiversity. Furthermore, Canada announced plans to phase out certain neonicotinoids gradually. Other countries, like Italy, Slovenia, and Switzerland, have implemented partial bans on neonicotinoids to protect bee populations. Such measures underline the increasing global awareness of pollinator health and environmental sustainability.

The length of neonicotinoid persistence in plants is a clear advantage for farmers and people seeking protection against insects. However, long-term protection can also be seen as a disadvantage, since it increases the facilitates of neonicotinoids entering the environment through means such as pollen and nectar carried by animals, or even the water cycle. The significant amount of their residues in the environment is a consequence of their intensive use over many years, posing a concern for humans, animals, and the environment alike. Given this, it is crucial to have strict monitoring of their content, necessitating determination methods with appropriate sensitivity and selectivity (Selahle et al. 2021). The responsibility for addressing this oversight lies within the domain of chemical analysts. Their role extends beyond the quantitative and qualitative assessment of pesticide levels in the environment. It also involves monitoring other hazardous substances that can contaminate water, soil, and air through various pathways stemming from industries such as metallurgical, chemical, pharmaceutical, agricultural, and heating (e.g. heavy metals Suiyi et al. 2024; Sýs et al. 2017), H_2_S (Lu et al. 2023), N_2_H_4_ (Lu et al. 2024), SF_6_ (Wang et al. 2023)). Consequently, the health and well-being of our environment depend significantly on accurate chemical analysis. However, to achieve the required results, the proper selection and application of all stages of chemical analysis is mandatory.

The sample preparation process plays an important role in achieving the desired sensitivity and selectivity, when analysing neonicotinoid pesticides. To date, numerous sample preparation methods for analysis have been developed. These include liquid–liquid extraction (Timofeeva et al. 2017), liquid phase microextraction (Vichapong et al. 2013, 2019; Kachangoon et al. 2020a, 2020b), solid-phase extraction (Wang et al. 2020b; Zaidon et al. 2019; Ghiasi et al. 2020), QuEChERS (Paradis et al. 2014; Tanner and Czerwenka 2011), and anion exchange-disposable pipette extraction (Song et al. 2018). Following sample preparation, the next essential step is selecting the appropriate analytical method. In recent years, numerous methods for the trace determination of neonicotinoids in various sample matrices have been documented. These include primarily gas chromatography (Shamsipur et al. 2016; Amelin et al. 2012), liquid chromatography, and high-performance liquid chromatography techniques in combination with various types of detectors, such as DAD (Selahle et al. 2020), mass spectrometry (Lu et al. 2020), tandem mass spectrometry (Gaweł et al. 2019), and high-resolution mass spectrometry (Moreno-González et al. 2018). Additionally, methods like immunosorbent assays (Liu et al. 2016), capillary electrophoresis (Nasiri et al. 2020), thermal lens spectrometry (Guzsvány et al. 2007), fluorimetry (Vílchez et al. 2001), or Fourier transform infrared spectroscopy (Quintas et al. 2004) have also been explored.

However, the above-mentioned analytical methods, despite providing accurate and reliable results, are relatively expensive, time-consuming, and require experience due to their complexity. In response, there has been an effort to develop new methods suitable for in situ research that is less expensive and difficult to implement. Electrochemical methods emerged as a solution, capable of determining not just neonicotinoid insecticides but a wide range of other substances. This includes organic compounds like drugs (Rauf et al. 2005; Özcan and Şahin 2007), proteins (Herzog and Arrigan 2007), vitamins, and hormones (Zhang and Jiang 2005), as well as inorganic species like heavy metals, e.g. mercury and lead (Jovanovski et al. 2015; Jedlińska et al. 2018). These methods offer significant advantages due to their sensitivity and selectivity, making them valuable tools in analytical chemistry and environmental monitoring. Electrochemistry is characterized by low cost, flexibility, and the possibility of field testing, which turned out to be a great alternative to traditional and time-consuming chromatographic methods (Lezi and Economou 2015; Selahle et al. 2021). The above-mentioned features of electroanalysis suggest that electrochemical methods significantly develop the possibilities of chemical analyses in many aspects of human activity, including clinical analyses, environmental monitoring, and food testing. Presently, a primary objective in electrochemical research is to reduce the volume of samples and develop systems for portable data analysis.

Neonicotinoids have been widely used for combatting insect pests in crops. However, with extended usage, it became evident that certain neonicotinoids adversely impact the environment. This review aims to provide a comprehensive overview of the electrochemical methods used to determine neonicotinoids. Our examination of electrochemical determination methods is categorized based on the electrochemical procedure, the type and modification of the electrode, and the observed electrode mechanism. By synthesizing and analysing the existing research, our review provides invaluable insights and guidance to researchers, shedding light on the current development of neonicotinoid electroanalysis and its potential future perspectives.

## An overview of the electrochemistry of neonicotinoids

Electrochemical studies have introduced new possibilities for the determination of substances that provide very high sensitivity and selectivity. These methods are relatively inexpensive, fast, and a little complicated (Hoyos-Arbeláez et al. 2017). Such methodologies have also been employed to detect trace amounts of neonicotinoids. Due to the variety of electrochemical methodologies for the determination of neonicotinoid insecticides, this paper will summarize them according to the type of used technique, the electrode material and its modification, and the observed electrode mechanism. Collected and summarized analytical parameters are available also in the Online Resource 1.

### Types of electrochemical techniques used

#### Cyclic voltammetry

**Fig. 2:**
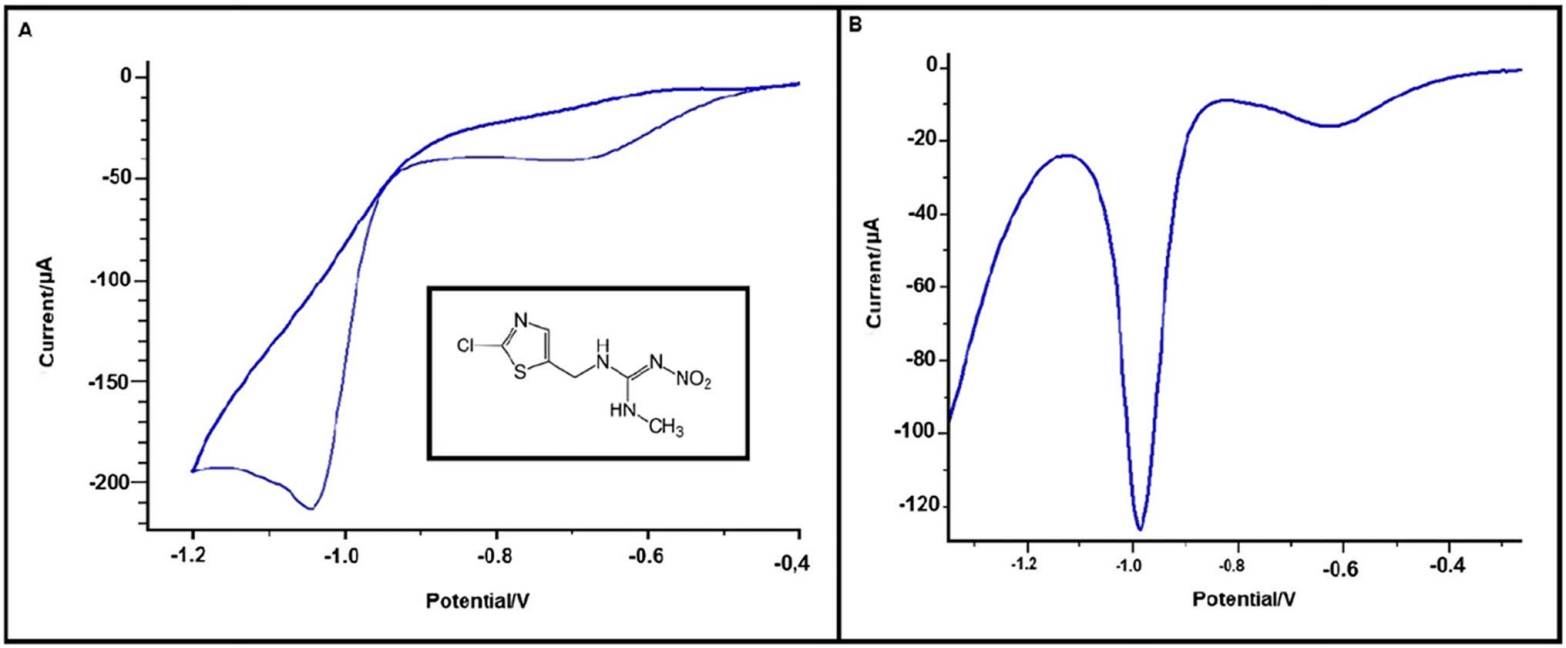
Cyclic (**A**) and differential pulse voltammogram (**B**) represent the electrochemical behaviour of 10^−3^ M clothianidin in pH 6 BR buffer solution at bare PGE. The potential sweeps applied between − 0.4 and − 1.2 V with the scan rate 20 mV s^−1^. Reproduced from Öndeş and Muti (2020) with permission

Cyclic voltammetry (CV) is one of the basic techniques used in electrochemical measurements. It might be used in the case of neonicotinoids as well, e.g. for the electroreduction of imidacloprid (Guzsvany et al. 2005; Lei et al. 2013, 2014; Paiva et al. 2018; Chen et al. 2013; Zhangsun et al. 2022) as well as determination of thiamethoxam (Zhangsun et al. 2022; Kumaravel and Chandrasekaran 2012) and dinotefuran (Zhangsun et al. 2022). Despite its widespread use, CV exhibits relatively lower sensitivity compared to other voltammetric methods. However, it is still applied for detecting trace amounts of neonicotinoids. A summation on the crucial details of each study is given in Online Resource 1. Researchers typically opted for the electrochemical reduction of the compounds they studied, employing varied potential ranges depending on the electrode material, usually starting from potential close to 0 V (Guzsvany et al. 2005; Lei et al. 2013; Zhangsun et al. 2022; Kumaravel and Chandrasekaran 2012) or using most of the available potential window for particular electrode material (Paiva et al. 2018). Scan rates, an essential parameter in CV, vary among studies, with 0.1 V s^−1^ being the most frequently used (Lei et al. 2013, 2014; Chen et al. 2013; Kumaravel and Chandrasekaran 2012). Supporting electrolytes, predominantly buffer solutions such as Britton-Robinson (BR) and phosphate (Ph), are commonly utilized, with a near-neutral pH range of 6.8 to 7.3. Notably, the pH of the supporting electrolyte significantly influences measurement outcomes, as demonstrated by Kumaravel et al.’s study on thiamethoxam, which utilized a pH 2.0 BR buffer (Kumaravel and Chandrasekaran 2012). Figure 2A depicts the electrochemical behaviour of clothianidin in BR buffer with pH 6 (Öndeş and Muti 2020).

#### Linear sweep voltammetry

Linear sweep voltammetry (LSV), akin to CV, is another basic voltammetric technique. The basis of the LSV technique can be considered as a one-direction scan, similar to half of a CV scan. This method has been used to detect trace amounts of neonicotinoids in artificial matrices and real samples. However, despite its simplicity and practicality, LSV’s sensitivity remains moderate compared to more advanced techniques. For a consolidated summary of the analytical details extracted from all studies, please consult Online Resource 1. Interestingly, Zhang et al. determined trace amounts of the fourth-generation neonicotinoid, paichongding, using LSV (Zhang et al. 2016) and being the only one electrochemical report for this compound so far. Again researchers predominantly employ LSV for the reductive determination of neonicotinoid insecticides, e.g. Liu et al. used negative potentials ranging from − 0.7 to − 1.4 V to determine imidacloprid (Liu et al. 2014), whereas determination of nitenpyram by Zhang et al., paichongding by Zhang et al., and thiamethoxam by Xie et al. was conducted in the same potential ranges, namely from − 0.2 to − 1.2 V (Zhang et al. 2017, 2016; Xie et al. 2017). Variations in measurement parameters, such as potential ranges and buffer solutions, further contribute to the heterogeneity of results (Chi et al. 2022; Kong et al. 2013). While LSV offers a practical approach for neonicotinoid detection, careful consideration of experimental parameters is essential for meaningful interpretation of results and establishment of robust analytical methodologies.

#### Differential pulse voltammetry

Differential pulse voltammetry (DPV) is an advanced voltammetric technique in which potential pulses are applied to a linearly changing potential ramp. DPV, known for effective background current reduction, boasts high sensitivity (Bhavik 2021). This technique has found applications in determining trace amounts of various compounds such as acetamiprid (Jin et al. 2016; Harery et al. 2023), clothianidin (Öndeş and Muti 2020; Guzsvány et al. 2011; Silva et al. 2023), imidacloprid (Lei et al. 2013; Chen et al. 2013; Majidi and Ghaderi 2017; Zhao et al. 2017; Guzsvány et al. 2011; Majidi et al. 2011, 2016; Pan et al. 2016; Oliveira Fernandes et al. 2024), nitenpyram (Wang et al. 2020a), dinotefuran (Mei et al. 2024), and thiamethoxam (Kumaravel and Chandrasekaran 2012; Guzsvany et al. 2006; Papp et al. 2010). Taking into account the number of compounds and the works found on the electrochemical determination of neonicotinoids using the DPV technique, it can be concluded that it presents a wide spectrum of possibilities for the determination of insecticides, as depicted in Online Resource 1. Notably with applying potential pulses, the DPV technique achieves lower detection limits than the CV and LSV techniques discussed above. In most reported studies, the reductive determination of neonicotinoids was the focus, with a single exception as Jin et al. developed a method for the photocatalytic degradation of acetamiprid through the oxidation of the target compound (Jin et al. 2016). Reported limits of detection (LOD) range from 3.3 × 10^−14^ to 3.19 × 10^−5^ mol L^−1^ for different neonicotinoids. Variations in measurement parameters, such as studied potential ranges, buffer type, and pH, as well as parameters describing construction of applied potential pulses, suggest rather empirical approach on the selection of optimal values being rather specified for a single neonicotinoid electrochemical detection (cf. Online Resource 1). Standardization of experimental protocols and parameters would be crucial for enhancing the reliability and robustness of DPV-based analytical methodologies. Figure 2B shows a differential pulse voltammogram, which captures the electrochemical behaviour of clothianidin in a pH 6 BR solution (Öndeş and Muti 2020).

#### Square wave voltammetry

Square wave voltammetry (SWV), a member of the electrochemical pulse techniques, is a high-amplitude specialized version of differential pulse technique. In SWV, a symmetrical square wave, superimposed on a fundamental step potential, is applied to the working electrode surface. The distinct advantage of SWV lies in its ability to sample the current twice during each square wave cycle (Wang 1994). SWV is characterized by one of the highest sensitivities and measurement speeds (Smarzewska et al. 2012). The method’s extreme usefulness arises from the fact that the net current exceeds components, e.g. forward or reverse current (Wang 1994), if only the studied redox mechanism is not totally irreversible. SWV combines the best features of pulse voltammetry and basic CV or LSV techniques; therefore, its detection limits can be compared with analytical methods such as chromatography or spectroscopy (Smarzewska et al. 2012; De Oliveira et al. 2016).

Owing to its pronounced sensitivity, SWV has been used extensively to determine trace amounts of neonicotinoid insecticides. Notable applications of the SWV technique include determining dinotefuran by Smarzewska et al. (Smarzewska et al. 2012), clothianidin by Guziejewski et al. (Guziejewski et al. 2011), imidacloprid by Bruzaca et al. (Bruzaca et al. 2021), and imidaclothiz by He et al. (2023). Brycht et al. used this technique to determine trace amounts of clothianidin, nitenpyram, and thiacloprid (Brycht et al. 2012). Thiamethoxam has also been determined by the SWV technique by Ajermoun et al. (Ajermoun et al. 2019), while Oliveira et al. included both imidacloprid and clothianidin (Oliveira et al. 2018). For a comprehensive summary of the analytical parameters from each study, please refer to Online Resource 1. These works primarily revolved around the reductive determination of neonicotinoids, and the potential windows used shared similarities, typically ranging from − 0.2 to − 1.6 V. The authors used their own, empirically determined, sets of SWV parameters, adapted to individual measurements. Despite that the values fall into the commonly used ranges, a careful selection of frequency and amplitude is needed each time. As for supporting electrolytes, buffered solutions were the go-to, with many authors opting for BR or phosphate buffers in a pH span from 6.0 to 10.4 (Smarzewska et al. 2012; Bruzaca et al. 2021; Brycht et al. 2012; Ajermoun et al. 2019; Oliveira et al. 2018; Guziejewski et al. 2011). In summary, SWV offers a powerful approach for the detection of neonicotinoid insecticides, owing to its high sensitivity and measurement speeds.

#### Other electroanalytical techniques

It is worth noting that other techniques, such as fast Fourier transform coulometric admittance voltammetry (FFTCAV) and amperometry, have also been used in the electrochemical determination of neonicotinoids. Both of them have been used for the reductive determination of thiamethoxam. While the amperometry technique showed a relatively low limit of detection at 8.8 × 10^−7^ mol L^−1^, it was FFTCAV that achieved best electrochemical results, recording one of the lowest LODs at the picomolar level: 6.2 × 10^−12^ mol L^−1^ (Kumaravel and Chandrasekaran 2012; Norouzi et al. 2017). It is worth highlighting the importance of exploring emerging techniques, such as electrochemical Faradaic spectroscopy (Jadresko et al. 2018) and differential square-wave voltammetry (Mirceski et al. 2019). These innovative approaches offer higher sensitivities compared to standard methods (Guziejewski et al. 2023), presenting promising avenues for advanced neonicotinoid detection methodologies.

### Types of the electrode used and its modifications

The efficiency of electrochemical measurements is heavily influenced by the choice of an appropriate working electrode. Historically, mercury electrodes were the preferred choice because of their significant advantages: high repeatability, a low background current, and a wide cathodic potential window. Moreover, the ability of mercury to form amalgams with most metals expanded its applicability in numerous applications. However, due to its status as a heavy metal, mercury is highly toxic and can bioaccumulate across many organisms (Sánchez-Calvo et al. 2020; Serrano et al. 2016). Therefore, for environmental safety reasons, the use of mercury electrodes in such quantities as before was discontinued and the search for equally efficient but environmentally safe electrodes began and is still continued. Currently, the most commonly used electrodes are glassy carbon electrodes (GCE), which are not only environmentally safe, but also characterized by low production costs, excellent electrical conductivity, and high hardness. Moreover, these electrodes are impermeable, electrochemically inert in a wide potential window, and are easy to modify with their working surface (Abdel-Aziz et al. 2022). These advantages make glassy carbon electrodes highly popular in electrochemical research and have also found their wide use in the determination of trace amounts of neonicotinoids. Other electrodes used for the determination of these compounds are the carbon-ceramic electrode (CCE), carbon paste electrode (CPE), pencil graphite electrodes (PGE), metallic silver electrode (MSE), and the afore-mentioned mercury-based electrode.

#### Mercury-based electrode

**Fig. 3:**
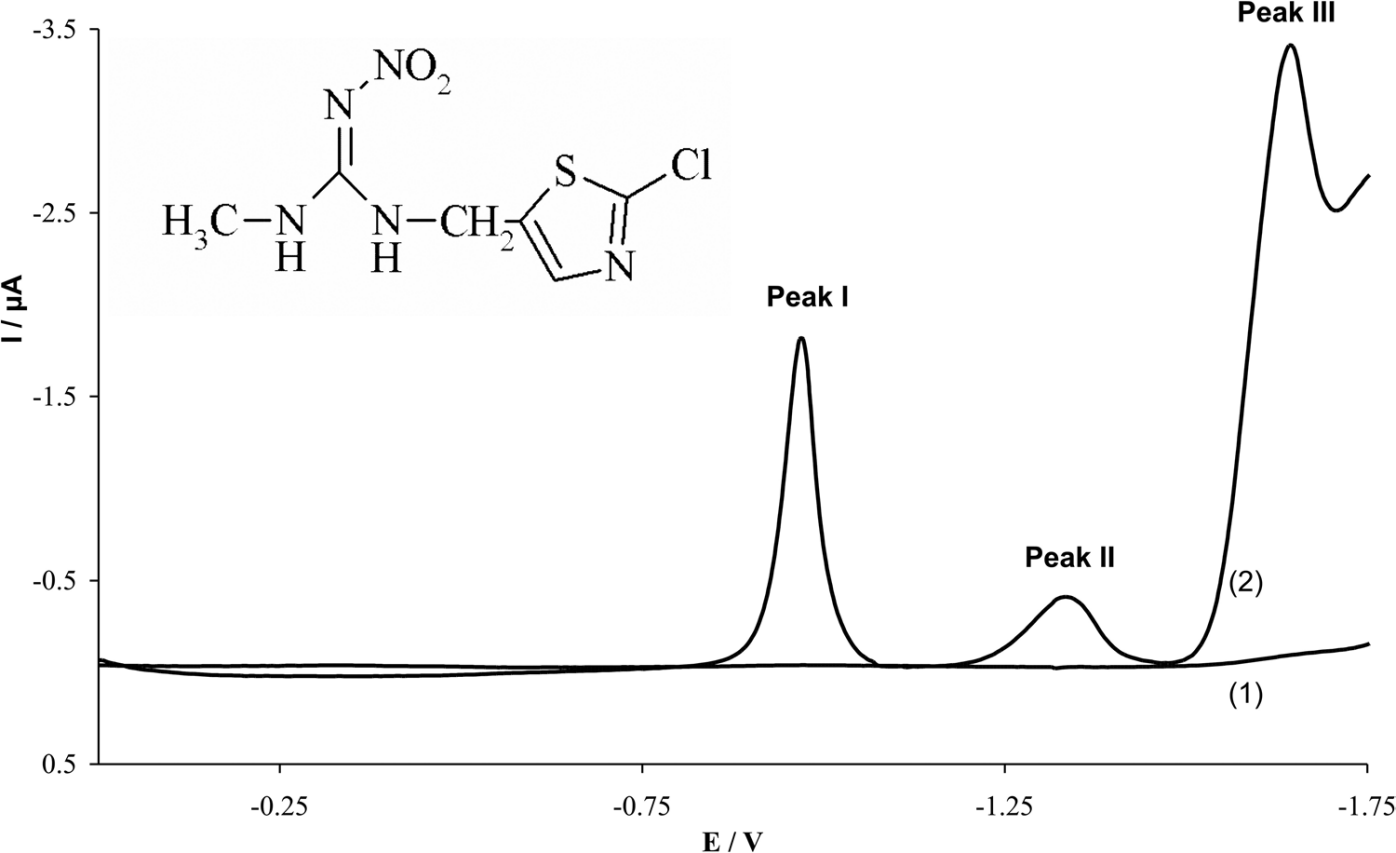
SW CSV voltammogram of 3 × 10^**–**6^ M clothianidin in BR buffer pH 8.1; conditions: frequency 50 Hz, amplitude 25 mV, step potential 5 mV, *t*_acc_ = 30 s at 0 V. Blank (1), added clothianidin (2). Inset: clothianidin structure. Reproduced from Guziejewski et al. (2011) with permission

Three distinct approaches for determining neonicotinoids using mercury electrodes have been reported. Electroanalysis was carried out using two different types of electrodes. The first method employed a hanging mercury drop electrode (HMDE) to determine trace amounts of clothianidin (Fig. 3) in water samples (Guziejewski et al. 2011). The other two methods used a renewable silver amalgam film electrode (Hg(Ag)FE) for the determination of dinotefuran, clothianidin, nitenpyram, and thiacloprid (Smarzewska et al. 2012; Brycht et al. 2012).

The use of sensitive and repeatable mercury electrodes enabled the achievement of relatively low limits of determination for individual compounds. Online Resource 1 provides a concise overview of the key findings from each study. The use of renewable silver amalgam film electrodes resulted in higher detection limits but reduced the consumption of toxic mercury during tests. This adaptation offers safer work practices, mitigating adverse environmental impacts while maintaining relatively low determination limits. Moreover, all the authors who used mercury electrodes successfully applied their findings to real samples, such as water and carrot juice.

#### Carbon-ceramic electrode

Ceramic-carbon electrodes were first developed and introduced by Gun et al. (Gun et al. 1994) in response to the growing demand for enhanced sensitivity and speed of electrochemical reactions. These electrodes offer distinct advantages, notably the marriage of properties from carbon electrodes—such as the conductivity of carbon materials—with those of the sol–gel process, primarily a large specific surface area. The introduction of CCE electrodes led to the creation of a renewable electrode with a porous surface akin to carbon paste electrodes but with greater durability and stability (Módolo et al. 2013).

In the realm of neonicotinoid detection, two studies have explored the utilization of CCE electrodes and their modifications. Both studies focused on detecting imidacloprid in tomato samples (Majidi et al. 2011), with one extending its analysis to commercial preparations (Majidi et al. 2016). Majidi et al. developed an electrochemical sensor featuring copper(II) phthalocyanine (CuPc) deposited on the CCE electrode. Using cyclic voltammetry (CV), they demonstrated that the presence of CuPc modifications on the CCE surface produced a distinct reduction peak for imidacloprid at a potential of − 1.1 V. In contrast, without the CuPc modifier, only a small peak was observed at − 1.3 V. Consequently, the CuPc modification induced a shift in the reduction potential of the insecticide and amplified the peak’s height.

Moreover, the literature documents the modification of CCE electrodes with an ionic liquid, specifically 1-allyl-3-methylimidazolium tetrafluoroborate ([AMIM][BF_4_]). Scanning electron microscopy (SEM) and energy-dispersive X-ray analysis revealed the formation of graphene-like nanoplates on the surface of the modified CCE electrode. This combination, along with the high conductivity of the ionic liquid, facilitated sensitive studies on the reduction of imidacloprid, achieving a limit of detection of 3.1 × 10^−8^ mol L^−1^. The study demonstrated that modifying the CCE electrode with [AMIM][BF_4_] leads to high selectivity, sensitivity, and repeatability in studies focused on the reductive determination of imidacloprid. The proposed methodology was validated, with results consistent with those obtained via high-performance liquid chromatography (Majidi et al. 2016).

#### Carbon paste electrode

A CPE is an electrode made of a mixture of carbon powder, primarily graphite, and a binder known as a pasting fluid (Adams 1958). Since its development, this electrode has gained high attention within the electrochemical community. This is due to the appropriately combined physicochemical and electrochemical properties, such as low background current, low resistance, a wide range of potentials as well as low price, ease of modification, and easy removal of the electrode’s surface layer (Švancara and Vytřas 1993; Švancara et al. 2009).

CPEs have also found their application in the determination of neonicotinoid insecticides. In one particular study was used as the working electrode for the photodegradation of acetamiprid to 6-chloronicotinic acid (6CNA), the last stable product of acetamiprid’s degradation. The authors reported a LOD of 2.0 × 10^−10^ mol L^−1^. This LOD showcased a commendable correlation with the HPLC reference technique; further authors confirm the sensor’s effective performance in detecting acetamiprid within vegetable samples (Jin et al. 2016). Papp et al. proposed a research procedure based on the electroreduction mechanism of the nitro group using graphite powder and tricresyl phosphate (TCP), indicating that the latter substance can significantly reduce the amount of oxygen absorbed by graphite (Švancara and Vytřas 1993). The electrode’s surface remained unmodified leaving space for further improvements. The limit of detection was established at 1.2 × 10^−5^ mol L^−1^. The authors validated the methodology by determining the neonicotinoid in real-world samples, predominantly river water and commercial formulations and corroborated by the HPLC/DAD method (Papp et al. 2010). In another study, Guzsvány et al. proposed a method using the previously mentioned electrode based on tricresyl phosphate further modified with bismuth. By integrating bismuth powder into the carbon paste, the authors achieved an electrode modification that combines the advantages of both bismuth and CPEs. Consequently, the authors attained a detection limit of 6.65 × 10^−6^ mol L^−1^ (Guzsvány et al. 2011). In a separate study, Norouzi et al. introduced an advanced measurement technique termed FFTCAV, tailored for the determination of trace amounts of thiamethoxam and combined with a unique CPE. The result of this innovation was a carbon-ion electrode (CILE) characterized by its high electrical conductivity, increased reversibility, and high sensitivity. To further refine the electrochemical sensor’s attributes, the research team explored additional electrode modifications, e.g. gold nanoparticles (AuNPs), multiwalled carbon nanotubes (MWCNTs), and reduced graphene nanosheets (rGNs) attached to the electrode’s surface. These enhancements led to a substantial augmentation in both the surface area of the electrode and its electron transfer rate. As a consequence, the authors managed to develop a sensitive CPE-based sensor with a wide linear range and a low detection limit equal to 6.2 × 10^−12^ mol L^−1^ (Norouzi et al. 2017).

#### Pencil graphite electrode

Graphite, a common allotrope of carbon, is a unique substance because it exhibits both metal and non-metal properties. Moreover, it boasts high thermal and electrical conductivity, making it an exceptional material. The low cost of production and the ease of use and cleaning have made graphite electrodes very popular as working electrodes. Over time, PGE has demonstrated its attitude for analytical determinations, thanks to their pronounced sensitivity and consistent measurement repeatability (David et al. 2017). In a detailed study, Öndeş et al. introduced a method for the determination of trace amounts of clothianidin, as depicted in Fig. 2, based on the reduction mechanism of the insecticide. The methodology entailed an initial activation of the PGE, after which it was coated with a layer of single-walled carbon nanotube (SWCNT) by immersing it in the dispersion. Preliminary SEM analyses revealed that the surface of the modified PGE/SWCNT electrode was notably rougher than its bare PGE counterpart, signifying an augmented surface area for the modified version. A comparative analysis between the two electrodes showed that the PGE/SWCNT produced a signal approximately 1.8 times greater than its unmodified electrode. Capitalizing on this enhanced capability of the modified electrode, Öndeş et al. achieved a detection limit of 3.19 × 10^−5^ mol L^−1^ (Öndeş and Muti 2020).

#### Metallic silver electrode

**Fig. 4:**
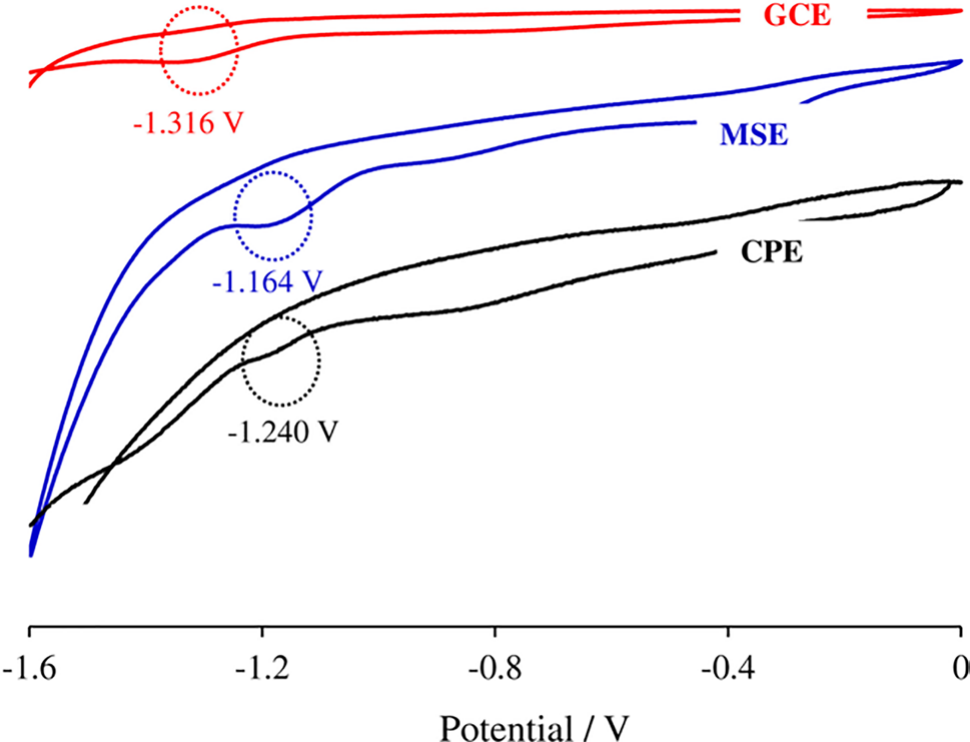
Cyclic voltammograms of the three electrodes, MSE, GCE, and CPE, in the potential range of 0 to − 1600 mV versus Ag/AgCl with a scan rate of 50 mV s^−1^, 1.0 × 10^−3^ mol L^−1^ of thiamethoxam in BR buffer (pH 10.4). Reproduced from Ajermoun et al. (2019) with permission

Ajermoun et al. proposed a novel method for the reductive determination of thiamethoxam, employing a MSE as the working electrode. Throughout their study, the research sought to contrast the efficacy of the MSE against other known electrodes, such as the GCE and the CPE. To provide evidence for the superiority of the MSE, the authors applied a range of techniques: CV, Tafel plots, and electrochemical impedance spectroscopy (EIS). The results, illustrated in Fig. 4, indicate that the MSE boasts greater electrocatalytic activity during the electroreduction of thiamethoxam compared to both the GCE and CPE. With further measurements carried out using the SWV technique, the authors identified a limit of detection at 5.49 × 10^−6^ mol L^−1^ and transferred the tests from artificial matrices to real-world samples, namely tomatoes and orange juices (Ajermoun et al. 2019).

#### Glassy carbon electrode

The GCE has been used by many authors to determine trace amounts of neonicotinoid insecticides. This is attributed to both its reduction and oxidation mechanisms and the ease of its surface modification. For a consolidated summary of the analytical details extracted from all studies, please consult Online Resource 1. A plethora of substances are available that can enhance its analytical capabilities. A straightforward approach to voltammetry involves the use of a bare electrode, devoid of any modifications. Such a methodology was proposed by Guzsvany et al. to ascertain the presence of two neonicotinoids: imidacloprid and thiamethoxam. The researchers leveraged the electroreductive mechanisms of the nitro groups found in these compounds and successfully applied procedure to real potato samples and commercial formulations. Alarmingly, their results unveiled that the potato samples contained double the permissible limit of neonicotinoid insecticides. Specifically, imidacloprid was present at 0.09 mg kg^−1^, whereas the acceptable threshold is 0.05 mg kg^−1^ (Guzsvany et al. 2005).

Graphene oxide (GO) is a modern material used for the modification of electrode surfaces. Characterized by its layered structure, GO incorporates a rich array of oxygen functional groups, including epoxides, alcohols, and carboxylic acids. GO is ideal for electrochemical applications, particularly in electrochemical sensors, because of its large surface area and excellent electrical conductivity. In addition, it can be used in combination with other modifiers to improve the sensitivity and selectivity of measurements. Therefore, it is currently one of the most frequently used surface modifiers of GCE (Lei et al. 2014; Oliveira et al. 2018; Zhang et al. 2017). Exploring this avenue, Lei et al. ventured into utilizing graphene oxide-modified (GCE/GO) to electrochemically determine imidacloprid concentrations. Expanding the applicability of their methodology, Lei et al. successfully deployed their method to ascertain trace amounts of imidacloprid in both lakes and tap water samples (Lei et al. 2014). Synergistic composites on the electrode surfaces often elevate the selectivity and sensitivity of the electroanalytical methods. Therefore, some of the authors have developed combinations of the reduced form of GO (rGO) with substances enhancing electrochemical properties, such as β-cyclodextrin (β-CD), water-soluble β-cyclodextrin polymer (β-CDP), poly(o-phenylenediamine) (PoPD), and polycarbazole (PCz). Synergistic composites have a beneficial effect, due to the combination of the advantages of the substances present at the electrode surface. The combination of GCE/rGO/β-CD and GCE/rGO/β-CDP has been used to determine substances such as clothianidin (Oliveira et al. 2018), imidacloprid (Chen et al. 2013; Oliveira et al. 2018), nitenpyram (Zhang et al. 2017), thiamethoxam (Oliveira et al. 2018), and the fourth-generation neonicotinoid, paichongding (Zhang et al. 2016). Incorporating polymers like PoPD and PCz with GCE/rGO provided possible determination of imidacloprid (Kong et al. 2013; Lei et al. 2013). These modified electrode surfaces not only amplified the electrode’s sensitivity but also enabled the determination of neonicotinoids in real-world samples such as honey, pollen, beeswax (Oliveira et al. 2018), rice (Zhang et al. 2017), grains (Zhang et al. 2016), and pears (Kong et al. 2013). In a couple of studies, Guzsvany et al. employed a bismuth-modified GCE surface for electroanalytical studies (Guzsvany et al. 2006; Guzsvány et al. 2011). Therefore, reductive mechanism of nitro groups in both clothianidin and thiamethoxam was explored. This methodology was successfully extended to real sample testing, specifically potato and maize and consistent with HPLC/DAD reference method (Guzsvany et al. 2006). Synergistic composites are intended to increase the electrocatalytic activity of measurement systems. In this context, Zhangsun et al. introduced the nitrogen-doped octahedral nanoporous NiCu (N/NiCu@C) carbon composite. By creating an electrochemical sensor using GCE/N/NiCu@C, they were able to detect trace amounts of the neonicotinoids: dinotefuran, imidacloprid, and thiamethoxam. The N/NiCu@C composite-modified electrode surface enhanced the diffusion between the electrolyte and the active site, significantly optimizing the electrocatalytic efficiency. Zhangsun et al. successfully used the proposed electrode on real samples, specifically analysing apple, tomato, and potato samples (Zhangsun et al. 2022).

Since their discovery, carbon nanotubes (CNTs) have gained significant attention due to their large surface area, chemical stability, and outstanding electrical conductivity. These properties enable the amplification of analytical signals in electroanalytical chemistry (Bandaru 2007; Wang et al. 2006). Specifically, multiwalled carbon nanotubes (MWCNTs) have proven effective as single modifiers (Paiva et al. 2018), or they can form synergistic composites with other materials to further increase the sensitivity and selectivity of measurements (Liu et al. 2014; Zhao et al. 2017; Norouzi et al. 2017; Bruzaca et al. 2021). Paiva et al. proposed a single GCE surface modifier using functionalized MWCNT for determining trace amounts of imidacloprid in both artificial models and water samples (Paiva et al. 2018). Complex synergistic composites using multiwalled carbon nanotubes to modify the GCE surface have been also proposed by Liu et al., Zhao et al., and Bruzaca et al. (Liu et al. 2014; Zhao et al. 2017; Bruzaca et al. 2021). In a study by Liu et al., an electrochemical sensor was developed using polyaspartate acid (ASP) to anchor the MWCNT layer onto the GCE surface, resulting in a synergistic effect within the composite, and leading to a significant increase in the analytical signal for the tested pesticide. Moreover, the authors validated the efficacy of the proposed analytical method with satisfactory outcomes on real samples, primarily plants (Liu et al. 2014). Zhao et al. performed the task of determining imidacloprid using a hydrophilic ionic liquid monomer, specifically 1-(α-methylacrylate)-3-vinyl imidazolium bromide. The material obtained by the authors was characterized by a mesh-like structure, as well as a large electroactive surface area and adsorption capacity. However, Zhao et al. reported the highest electrochemical response to neonicotinoid presence*,* when the composite was additionally combined with functionalized graphene. Finally, the authors successfully employed the methodology on real samples, specifically cabbage and peeled apples, introducing a new method for the electrochemical determination of imidacloprid (Zhao et al. 2017). Bruzaca et al. proposed another modification employing MWCNT on a glassy carbon electrode. In their approach, they developed an electrochemical sensor that integrates functionalized multiwalled carbon nanotubes with the addition of Nafion. Furthermore, the authors successfully used the proposed sensor in studying samples of tap water, melon, and shrimp (Bruzaca et al. 2021). Wang et al. developed a method to determine nitenpyram by harnessing the synergistic effects of a binary nanohybrid comprising hydroxylated multiwalled carbon nanotubes (HCNT) and single-walled carbon nanohorns (CNH). By skilfully amalgamating suitable electrode surface modifiers with the inherent properties of the GCE, Wang et al. managed to achieve a LOD for nitenpyram at the nM range, specifically clocking in at 4.00 × 10^−9^ mol L^−1^. Furthermore, they adeptly applied this innovative method to discern trace quantities of nitenpyram in both crops and river water samples (Wang et al. 2020a). Silva et al. introduced an electrochemical sensor sensitive to detect trace amounts of the third-generation neonicotinoid, clothianidin. The foundation of this sensor is a GCE and the surface has been modified using carbon black (CB) materials combined with nanoparticles of the ternary SiAlSn oxide (SiO_2_/Al_2_O_3_/SnO_2_). The introduction of this specific SiAlSn oxide with CB as an electrode surface modifier enabled Silva et al. to achieve a desirable synergistic outcome. This synergy expanded the electrode’s electroactive surface, enhancing the overall sensor surface and markedly amplified the detection efficiency for the clothianidin. The efficacy of this electrochemical sensor was further validated by Silva et al. as they successfully used it to determine neonicotinoid levels in real-world samples like tap water and apple juice (Silva et al. 2023).

**Fig. 5:**
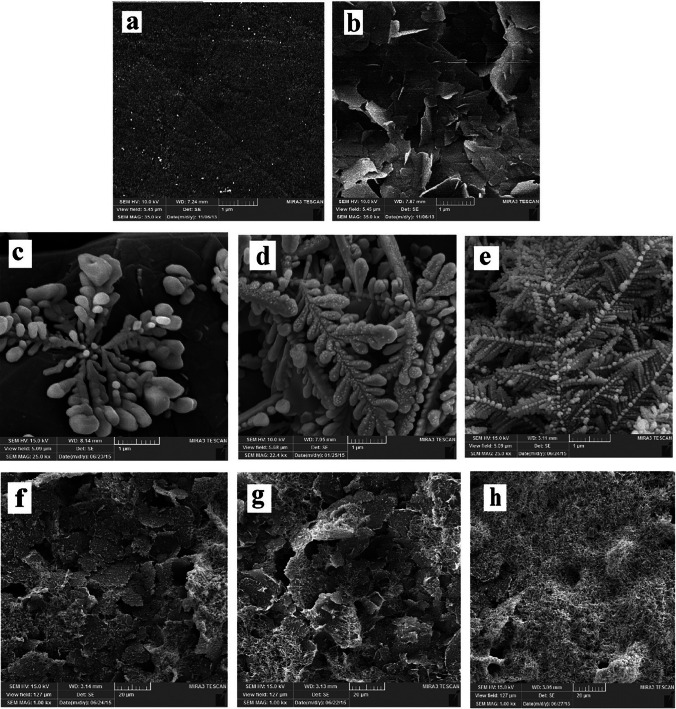
SEM images of (**a**) bare GCE, (**b**) GNs/GCE, and AgNDs/GNs/GCE synthesized under applied time of 120 s and applied potential of (**c**) + 0.1 V, (**d**) − 0.1 V, (**e**) − 0.3 V, AgNDs/GNs/GCE synthesized under applied potential of − 0.3 V and applied time of (**f**) 60 s, (**g**) 120 s, and (**h**) 180 s. Reproduced from Majidi and Ghaderi (2017) with permission

Silver is well known and widely used in the electrochemistry environment due to its highest electrical conductivity among metals. Advancements in nanotechnology have facilitated the synthesis of metal and oxide nanoparticles, which can advantageously modify electrode surfaces. The combination of silver presence with the possibilities offered by nanotechnology led to the synthesis of silver nanoparticles (AgNP) with a strong electrocatalytic effect, which is very desirable in electrochemical measurements (Kumaravel and Chandrasekaran 2012). Numerous studies have documented the application of silver nanoparticles as electrode modifiers in the determination of neonicotinoids. For instance, Pan et al. introduced an eco-friendly and cost-effective method to synthesize silver nanoparticles for detecting imidacloprid. Furthermore, the proposed sensor—built on a GCE modified with silver nanoparticles—demonstrated its efficacy by accurately identifying neonicotinoid residues in real soil samples (Pan et al. 2016). Majidi et al. proposed a method for imidacloprid detection that capitalized on the synergistic interplay between silver nanodendrimers (AgNDs) and graphene nanosheets (GNs, cf. Figure 5). This modified electrode exhibited remarkable electrocatalytic traits for the reductive determination of imidacloprid, underlining its high repeatability and sensitivity (Majidi and Ghaderi 2017). Kumaravel et al., on the other hand, presented a method for thiamethoxam detection that utilized a GCE surface modified with silver nanoparticles, further enhanced by sodium dodecylsulfate (SDS) (Kumaravel and Chandrasekaran 2012). Moreover, both Majidi et al. and Kumaravel et al. successfully transferred the performance of electrochemical sensor methodologies to real samples, specifically cucumber and potato, respectively (Majidi and Ghaderi 2017; Kumaravel and Chandrasekaran 2012).

#### Polymer-based electrode materials

Modern electrochemistry is marked by a wide spectrum of sensors that prioritize simplicity, reliability, repeatability, analysis speed, high sensitivity, ease of use, and, importantly, cost-effectiveness (Mei et al. 2024). While traditional electrodes remain relevant, many contemporary electrochemical sensors are predominantly composed of specially crafted polymer materials (e.g. molecularly imprinted, 3D printed, electrospinned, laser-induced). These polymers are not only tailored for electrochemical applications but also desired in purification, separation, drug delivery, boast traits such as biodegradability, chemical and mechanical stability, heightened selectivity and sensitivity, and environmental safety etc. (Harery et al. 2023).

**Fig. 6:**
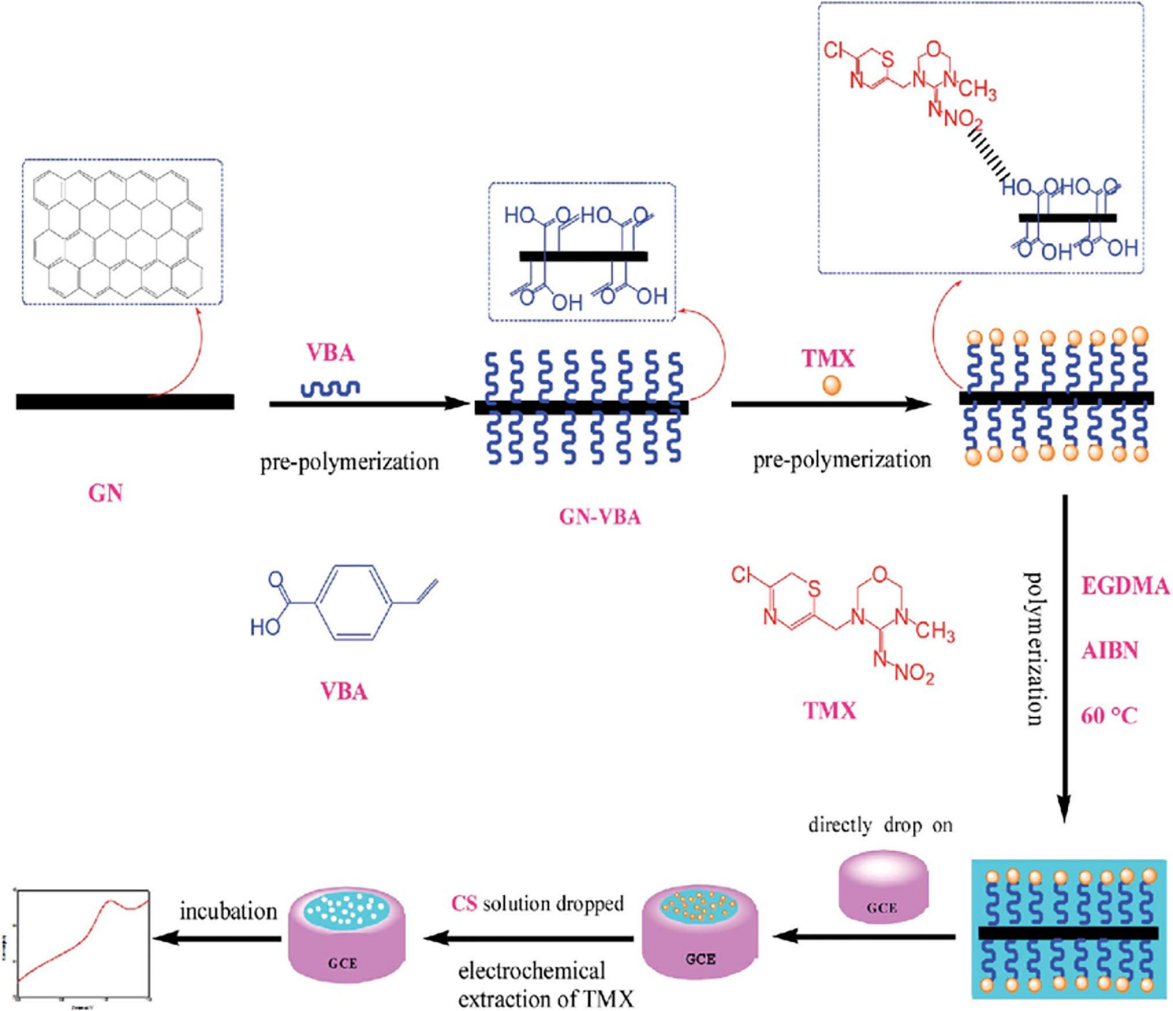
Schematic diagram of the procedure for the preparation of MIP-GN/GCE and concept for the selective electrochemical detection of thiamethoxam. Reproduced from Xie et al. (2017) with permission

Various studies have explored the modification of a GCE with polymers (Fig. 6), specifically a composite of p-vinylbenzoic acid (MIP) and graphene (GN), as well as poly(L-ornithine, PLO) for detection of thiamethoxam. For instance, Xie et al. successfully streamlined the electrode surface modification process using GCE/MIP/GN electrodes, while preserving the distinctive properties of the modified electrode. The resultant electrochemical sensor, equipped with an ultra-thin foil, bolstered electron transfer capability and heightened detection sensitivity. Conversely, Chi et al. devised an environmentally benign electrode modification characterized by its robust stability and consistency. Furthermore, both studies successfully validated their findings by applying the developed sensors for real sample analyses, notably in brown rice and commercial formulations (Xie et al. 2017; Chi et al. 2022). Recent advancements in electrochemistry have enabled the determination of imidaclothiz, a fourth-generation neonicotinoid. He et al. proposed a method based on a sensor composed of GCE modified with zeolitic imidazolate framework-8 and polyaniline (GCE/ZIF-8/PANI). The introduction of highly conductive PANI in the system significantly improved the sensor conductivity, increasing the low peak currents associated with the poor conductivity of ZIF-8. Additionally, He et al. validated the effectiveness of the proposed method for determining imidaclothiz in vegetable samples, achieving recovery rates ranging from 91.10 to 104.25% with a RSD of 4.26% (He et al. 2023). Electrochemical sensors employing 4-vinyl pyridine (MIP technology), 3D-printed conductive fibres crafted from polylactic acid biopolymers, and polycaprolactone were used to determine trace amounts of acetamiprid, dinotefuran, and imidacloprid, respectively. Each sensor exhibited exceptional sensitivity (up to 10^−14^ mol L^−1^), selectivity, and repeatability. Furthermore, the feasibility of employing these methods with readily available drug and food samples was affirmed by all authors (Harery et al. 2023; Mei et al. 2024; Oliveira Fernandes et al. 2024).

### Type of electrode mechanism

The utilization of redox mechanisms for the determination of compounds within the neonicotinoids family is closely related to the presence of characteristic groups previously discussed in this work. Neonicotinoids that belong to the N-nitroguanidine group display higher polarity and increased chemical reactivity; crucially, they are easily reducible. As a result, methodologies to detect their residues are primarily based on the nitro group’s reduction mechanism. Conversely, compounds within the N-cyanamidine group exhibit lower reactivity, attributed to the absence of an oxygen atom in their structure. Hence, methods for determining this group of neonicotinoids largely focus on oxidation mechanisms. The differing approaches among these groups dictate varied approaches and methodologies when pinpointing trace amounts of these compounds.

#### Reductive mechanisms

**Fig. 7:**
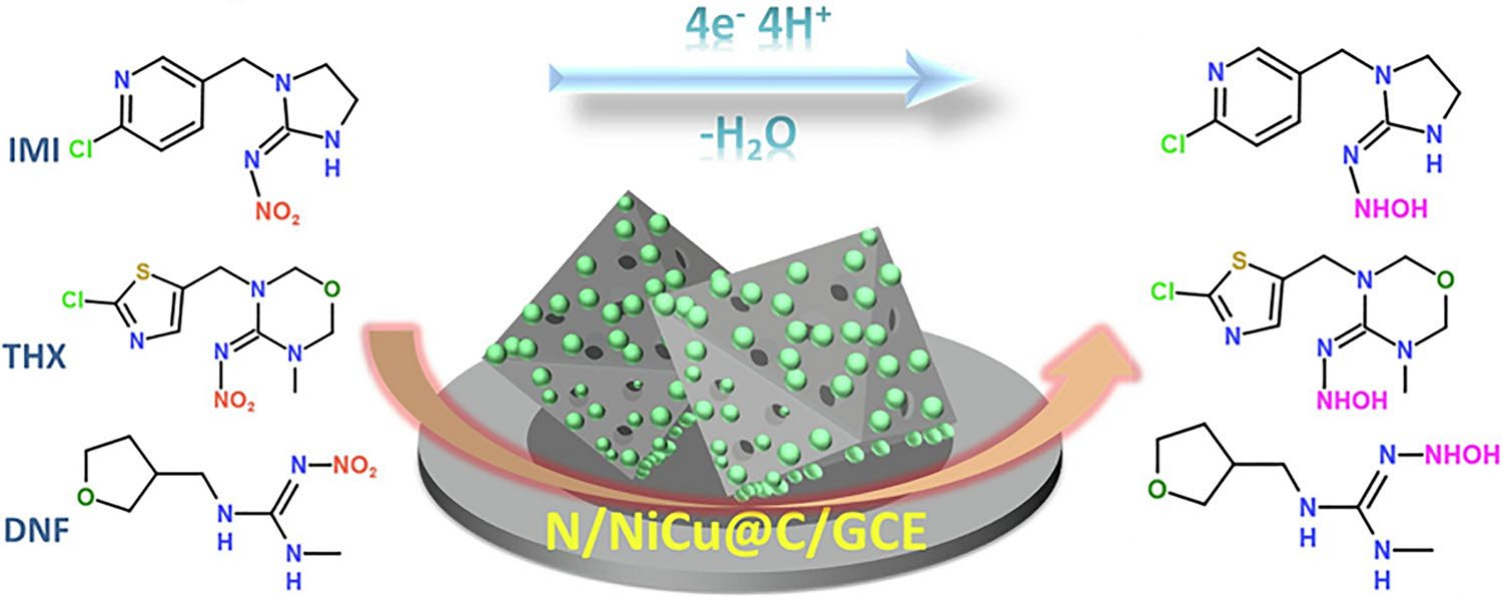
The mechanism of electrocatalytic reduction of imidacloprid, thiamethoxam, and dinotefuran at the N/NiCu@C/GCE. Reproduced from Zhangsun et al. (2022) with permission

**Fig. 8:**
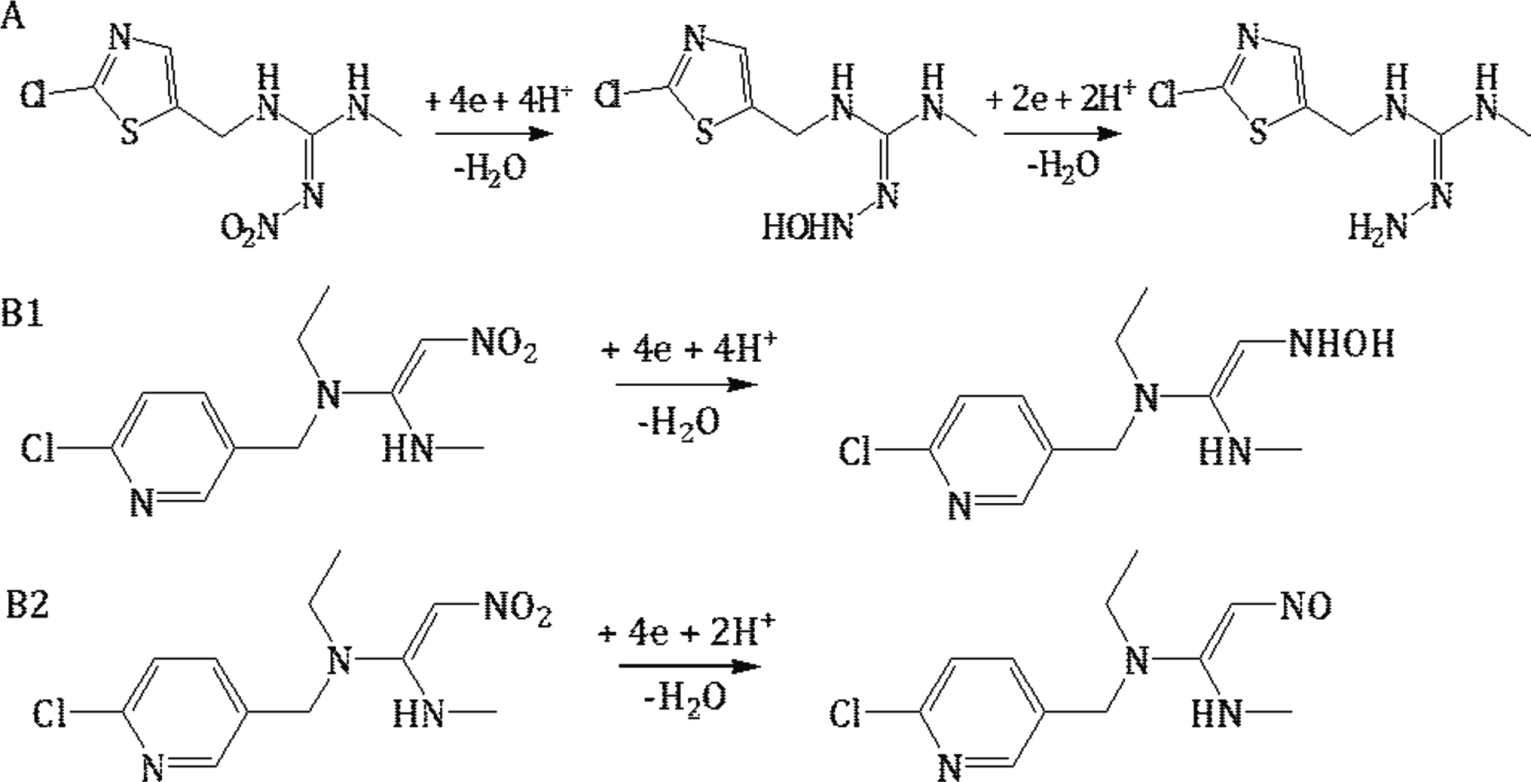
The proposed schematic electroreduction mechanism of (**A**) clothianidin, (B1) nitenpyram in pH < 9, and (B2) nitenpyram in pH > 9

The reduction mechanism of the nitro group has been extensively leveraged by researchers to determine trace amounts of N-nitroguanidine neonicotinoids. For such compounds, including imidacloprid, thiamethoxam, nitenpyram, dinotefuran, and clothianidin, the reduction mechanism follows an irreversible two-step sequence. The initial phase involves a 4-electron reduction of the nitro group (− NO_2_) to the hydroxylamine group. Concurrently, four protons are exchanged (Figs. 7 and 8). Subsequently, this hydroxylamine group undergoes reduction to form the pertinent amino group (Fig. 8). This second step of reduction involves the exchange of two electrons The specific outcome of the reduction process, i.e. the emergence of one or two peaks within a notably negative potential range, depends on both the experimental conditions and the nature of the neonicotinoid compound in question (Guziejewski et al. 2011; Öndeş and Muti 2020; Smarzewska et al. 2012; Bruzaca et al. 2021; Majidi and Ghaderi 2017; Zhao et al. 2017; Guzsvany et al. 2005, 2006; Guzsvány et al. 2011; Majidi et al. 2011, 2016; Lei et al. 2013, 2014; Pan et al. 2016; Paiva et al. 2018; Chen et al. 2013; Kong et al. 2013; Zhang et al. 2017; Wang et al. 2020a; Brycht et al. 2012; Xie et al. 2017; Ajermoun et al. 2019; Chi et al. 2022; Zhangsun et al. 2022; Kumaravel and Chandrasekaran 2012; Papp et al. 2010; Oliveira et al. 2018; Silva et al. 2023; Mei et al. 2024). However, it is noteworthy that for certain compounds, such as clothianidin and dinotefuran, there is a reported presence of an additional third peak in the realm of negative potentials. This third peak’s existence is attributed to the electrocatalytic evolution of hydrogen, a result of the guanidine group inherent in these compounds (Guziejewski et al. 2011; Öndeş and Muti 2020; Smarzewska et al. 2012; Mei et al. 2024).

The authors observed that the reduction process for nitenpyram can significantly differ based on the pH conditions, as delineated in Fig. 8. Specifically, Wang et al. indicated that at pH < 9, the nitro group undergoes reduction to hydroxylamine following the previously described electrode mechanism. However, when the pH > 9, the nitro group is reduced to the corresponding nitroso group. Interestingly, while the formation of the nitroso group involves the participation of four electrons in the reduction reaction—identical to the hydroxylamine formation—only two protons are exchanged in the reaction, when the pH is above 9 (Wang et al. 2020a).

Thiacloprid, a member of the neonicotinoids with the N-cyanamide group, lacks the − NO_2_ group. However, Brycht et al. suggested determining its residues via the reduction mechanism. In the potential range examined by the authors (− 1.1 to − 1.6 V), the neonicotinoid insecticide displayed a singular reduction peak. Furthermore, drawing from the E_p_–pH relationship, Brycht et al. reported that the ratio of exchanged electrons to protons is probably one-to-one (Brycht et al. 2012).

#### Oxidative mechanisms

The electrochemical determination of neonicotinoid residues using an oxidation mechanism has been reported solely in the studies of acetamiprid (Jin et al. 2016; Harery et al. 2023). Jin et al. used a photodegradation method, converting the neonicotinoid to 6-chloronicotinic acid (6CNA). This compound is believed to be the ultimate stable degradation product of acetamiprid. Both acetamiprid and 6CNA showed an oxidation peak at nearly the same potentials (around 0.81 V). The concurrent appearance of a peak at this potential suggests that acetamiprid undergoes photodegradation to 6CNA, validating its use for the indirect determination of the neonicotinoid (Jin et al. 2016).

#### Simultaneous determination of neonicotinoids

Although there is currently no evidence in the literature supporting simultaneous electrochemical analysis of multiple neonicotinoids in a single sample, the analysis of individual works on the electrochemical determination of these pesticides is encouraging. Most of the neonicotinoids discussed in this work belong to the group of N-nitroguanidines having the NO_2_ group in their structure. Consequently, all these compounds undergo similar redox reactions, in which the nitro group is reduced, yielding an electrochemical reduction signal at a comparable potential (the potential range for the entire group ranges from − 0.75 to − 1.6 V). The precise location of the tested reduction signal largely depends on the measurement system employed (such as the type of electrode, buffer, and its pH). With a properly selected system, the separation of reduction signals for individual compounds can be significant enough to allow for the simultaneous determination of multiple substances. One notable example is the study conducted by Brycht et al., in which clothianidin, nitenpyram, and thiacloprid were electrochemically determined. Although the first two compounds share the same functional group (-NO_2_), but exhibit reduction peak potential at − 1.0 and − 1.4 V, respectively (Brycht et al. 2012). Therefore, through the use of a properly selected electrode, along with an appropriate buffer and pH, the potential exists for simultaneous determination of several neonicotinoids in a single sample.

Furthermore, as demonstrated in this study, the electrochemical determination of acetamiprid relied on the mechanism of neonicotinoid oxidation. Therefore, a distinct signal at rather positive potentials (0.7 V) is recorded, as shown in the work by Jin et al. (2016). This suggests the potential for simultaneous determination of neonicotinoid substances using redox reaction mechanisms based on both oxidation and reduction. However, presented strategies for simultaneous neonicotinoid determination have not been experimentally validated and require further research.

## Conclusions

This review article sheds light on the significance of electrochemical methods in the determination of neonicotinoids, particularly in light of increasing concerns over their widespread use. Since the neonicotinoids market is one of the fastest-growing, it additionally proves the indisputable role these compounds play in controlling pests that threaten various agricultural and environmental ecosystems. However, their extensive use has precipitated a decline in nontarget organisms, particularly pollinators, insects, and birds. The potential chronic and sublethal effects on humans remain largely unknown. Although neonicotinoids are pivotal in contemporary agriculture, there is a conspicuous absence of thorough review articles in the literature, underscoring the pressing need for a consolidation of the extant electroanalytical knowledge on this subject. This material serves as a timely and comprehensive resource covering the application of electrochemical sensors for neonicotinoid detection. By examining different aspects, including electrode materials, techniques, and underlying mechanisms, this review aims to offer a comprehensive understanding of the electroanalytical approaches and challenges associated with them.

Moreover, it is important to acknowledge the increasing concerns and discussions around banning the use of certain neonicotinoids due to their potential adverse effects on beneficial insects and the overall ecosystem. It is noteworthy that many recently synthesized neonicotinoids remain underrepresented in existing literature, underlining the need for further analytical research on these compounds. With the great potential of sensitive electroanalytical techniques, there is an outstanding opportunity to utilize these techniques for identifying and quantifying neonicotinoids in environmental and food samples. Implementing these advanced techniques will undoubtedly enhance the scope and accuracy of monitoring and assessing neonicotinoid residues. Such advancements will, in turn, facilitate the development of effective strategies concerning their judicious application and regulatory oversight. The continued pursuit of sensitive, selective electroanalytical approaches is paramount, facilitating a deeper understanding of neonicotinoid behaviour, its potential risks, and its impact on various ecosystems. The combination of interdisciplinary collaborations with continuous research in this field will pave the way for sustainable and environmentally friendly pest management strategies, ensuring a healthier and balanced coexistence with nature. In light of the emerging environmental concerns, it is important to increase efforts towards exploring safer alternatives and mitigating the impact of neonicotinoids on biodiversity and ecological balance. We hope this review not only furnishes invaluable insights into the realm of electrochemical methodologies for neonicotinoid determination but also stimulates further research in this vital area of study.

## Data Availability

Data sharing is not applicable to this article as no new data were created or analyzed in this study.
